# Parents’ understanding and motivation to take part in a randomized controlled trial in the field of adolescent mental health: a qualitative study

**DOI:** 10.1186/s13063-020-04857-3

**Published:** 2020-11-23

**Authors:** Sally O’Keeffe, Katharina Weitkamp, Danny Isaacs, Mary Target, Virginia Eatough, Nick Midgley

**Affiliations:** 1 Child Attachment and Psychological Therapies Research Unit (ChAPTRe), Anna Freud National Centre for Children and Families, 4-8 Rodney Street, London, N1 9JH UK; 2 School of Health Sciences, City, University of London, London, UK; 3 University of Zurich, Zurich, Switzerland; 4 Adolescent and Young Adult Service, Tavistock and Portman NHS Foundation Trust, London, UK; 5 Research Department of Clinical, Educational and Health Psychology, University College London, London, UK; 6 Department of Psychological Sciences, Birkbeck College, University of London, London, UK

**Keywords:** Randomized controlled trial, Trial participation, Parents’ perspectives, Adolescence, Depression, Qualitative

## Abstract

**Background:**

Little is known about why parents agree to take part in randomized controlled trials for adolescent mental health. This study aimed to investigate parents’ perspectives on participating in a trial for psychological treatment of depression. The study explored parents’ motivations, understanding of the trial and perspectives on the acceptability of the trial.

**Methods:**

Sixty-five parents took part in this qualitative study. Their adolescent children had been randomly allocated to one of three active psychological treatments for depression as part of the IMPACT trial and were interviewed about their experiences of participating in the study. Semi-structured interviews were analysed using framework analysis.

**Results:**

For seven of the sixty-five parents, their experience of taking part in the trial was not covered in their interview so they were excluded from the analysis. The analysis was therefore based on the data from the parents of 58 adolescents taking part in the trial. The most commonly cited motivation for taking part in the study reported by parents was a desire to help others going through similar difficulties. Parents generally reported finding trial participation acceptable, although there were aspects that some reported finding less acceptable, including randomization and the burden of research assessments. Others spoke positively about the experience of trial participation and found it enjoyable or even therapeutic. Importantly, some did not appear to have a good understanding of the trial design, including randomization and treatment allocation.

**Conclusions:**

This study indicates that trial participation can be a positive experience for parents, yet it raises concerns about how trialists can ensure that consent is fully informed, given that some parents appeared to have a poor understanding of the trial. Future studies should seek to explore how communication with trial participants can be improved, to ensure that trial participation is fully informed. Patient and public involvement will be crucial in ensuring this communication is accessible to stakeholders.

**Trial registration:**

ISRCTN registry ISRCTN83033550. Registered on 15 October 2009

## Background

Randomized controlled trials (RCTs) are considered the ‘gold standard’ when it comes to evaluating the effectiveness of treatments. Such studies rely upon participation from service users, and in recent years, there has been growing emphasis on the inclusion of service users both in the design of such studies [1], and in order to better understand the barriers and facilitators to study participation [2, 3]. Many clinical trials now include a qualitative component as researchers have come to increasingly appreciate what qualitative methods can offer RCTs [4–6]. Qualitative research within trials has been used in a range of ways, including to explore the intervention being studied, the design and conduct of the trial, the outcomes and processes measured in the trial, the outcomes of the trial, and the health problems under study [5]. In the context of treatment for child and adolescent conditions, it is important to include the perspectives of both young people themselves and their caregivers—who may have different agendas, perspectives and ideas about what is and is not acceptable in the care of the child. The inclusion of parents in such studies is necessary as parents will often have a key role in psychological therapies for their children and in some cases may be actively involved in the sessions themselves [7] or maybe offered individual sessions alongside their child’s treatment [8].

In the context of treatment for mental health disorders in children and adolescents, there is a paucity of studies exploring their experiences of trial participation, including why young people decide to participate in clinical trials, and how well they understand the research process. In response to this, a previous study sought to explore the experience of young people participating in the Improving Mood with Psychoanalytic and Cognitive Therapies (IMPACT) trial, an RCT exploring the effectiveness of psychological therapies in the treatment of depression in young people, aged 11–17 [9, 10]. IMPACT-My Experience (IMPACT-ME) was a sibling study to IMPACT in which a sub-sample of the young people and parents taking part in the trial were interviewed about their experiences of being involved in the IMPACT trial [11]. Drawing on data from the IMPACT-ME study, a previous publication reported the adolescents’ experiences of taking part in the IMPACT trial [12]. Results showed that young people mostly spoke positively about participating in the trial and their interactions with the research assistants (who carried out six face-to-face outcome assessments with participants over an 18-month period), despite the lengthy assessments that the trial involved [12]. They spoke about motivations for taking part in the trial, including helping others as well as hoping it would have benefits for themselves, such as receiving superior treatment. However, concerns about the extent to which consent was truly informed were identified, as some young people seemed to have a limited understanding of the randomization process or the different treatment interventions [12]. Overall, trial participation was acceptable to these young people, but the previous analysis did not consider the experience of parents.

Parents’ perspectives on trial participation have been researched in trials of physical health problems. In such studies, considerations about whether to take part included safety and the practicalities around taking part [13]. Concerns about trial participation from parents included fear of losing control over their child’s treatment, while other parents were described as having a ‘nothing to lose’ attitude (p. 384, [14]). The extent to which these concerns and motivations for parents consenting for their child to take part in a clinical trial extend to the field of child mental health is unknown.

The present study seeks to complement the previously published study which explored adolescents’ experience of taking part in a trial for depression [12], by exploring the perspectives of the parents from the same sample as the previous study. Parents provide an important perspective as they will usually be the ones to decide whether to seek treatment for children under the age of 16, will be required to provide consent for their child to participate in an RCT and in many cases will also be involved in the treatment itself. Adolescents and their parents may well differ in terms of their understanding of a trial, their reasons for agreeing to participate and their perceptions of the acceptability of the trial. Yet, no studies have been reported which explore the parents’ experiences of taking part in an RCT in the field of child and adolescent mental health. This study seeks to address this gap in the literature—an important area for psychotherapy research, to ensure that studies are conducted in an acceptable and ethical manner and to help improve the design of future trials of psychological interventions involving young people and their parents.

### Aims

The aims of this study were (1) to explore parents’ motivations for participating in an RCT for adolescent depression; (2) to explore the understanding that parents had regarding a number of aspects of participation in the RCT, such as treatment options, randomization, differences between research and therapy, study design and understanding of the study’s objectives; and (3) to explore whether or not parents found participation to be acceptable and what affected this.

## Methods

### Setting for the study

This study involved secondary data analysis of interviews carried out as part of a larger longitudinal, qualitative study examining the experiences of a group of young people, their parents and therapists who were taking part in a randomized clinical trial investigating the treatment of adolescent depression.

#### Trial design

The IMPACT study was a large, multi-centre RCT conducted across three regions in the UK (North London, East Anglia and the North West), to investigate the effectiveness of three therapeutic approaches for adolescent depression [9, 10]. Participants were recruited from Child and Adolescent Mental Health Services (CAMHS), identified as potentially suitable by a clinician at the site. The inclusion criteria for the trial were that the adolescent was aged 11–17 years old and with a diagnosis of major depressive disorder according to DSM-IV criteria [16]. The exclusion criteria have been previously reported elsewhere [10]. Participants identified as eligible after a screening assessment were randomized to one of the three interventions, which were as follows:

i Cognitive behavioural therapy (CBT). Focused on identifying distorted cognitions and using explicit, shared goals, with an intended duration of up to 20 sessions delivered over 28 weeks [7].
j Short-term psychodynamic psychotherapy (STPP). Focused on giving meaning to the varieties of the young person’s emotional experiences and addressing difficulties in the context of the developmental tasks of the adolescent years, intended to be delivered over 28 weekly sessions [8].
k Brief Psychosocial Intervention (BPI). Focused on sleep hygiene, exercise and monitoring risk, with an intended duration of up to 12 sessions delivered over 20 weeks [17].

In total, 557 adolescents were screened for eligibility, and of those 465 adolescents met all the criteria and were recruited into the IMPACT trial. Of those recruited, they were randomized in relatively equal numbers to the three treatment arms (CBT *n* = 154, STPP *n* = 156, BPI *n* = 155). Adolescents received £15 as a thank you for each research meeting that they took part in.

#### Qualitative study

Linked to the IMPACT trial was its qualitative sibling study, IMPACT-My Experience (IMPACT-ME), which took place in the North London trial centre [11]. The IMPACT-ME study sought to explore the experiences of those taking part in the IMPACT trial in North London (*n* = 127). Due to resource issues, those participants in the other trial regions were not invited to take part in the IMPACT-ME study.

IMPACT-ME was a longitudinal study in which face-to-face interviews were conducted separately with the young people and their parents taking part in the IMPACT trial—before (baseline) and after therapy (36 weeks) and at 1-year follow-up (86 weeks). The baseline interviews were not included in the present study, as they did not explore the experience of the research as this was prior to the participant being randomized into the study. The present study draws on interviews conducted with parents at the post-treatment time points—i.e. 36 and 86 weeks, between October 2011 and January 2014. Of the 127 young people who took part in the IMPACT trial, where possible, their parents were invited to take part in the IMPACT-ME study. This was not possible for 43 young people who were aged 16 or 17 years old, who were taking part in their trial without the involvement of their parents. Of the remaining 84 where the parent had given consent to take part in the IMPACT trial, 19 parents did not take part in the IMPACT-ME study as they were unavailable or declined to be interviewed. In total, interviews were therefore collected with the parents of 65 young people taking part in the IMPACT trial.

In the IMPACT-ME study, separate versions of the interview schedules were used for adolescents and parents. These versions covered similar topics, while the parent version covered aspects of the experience specific to the experience of being a parent of an adolescent in the trial, e.g. concerning their own experiences as parents, both in relation to treatment and study participation. In a previous study, the experience of taking part in the trial from the perspective of adolescents was reported [12]. The present study is a complementary study reporting on the parents of those same cases.

#### Researcher characteristics and reflexivity

Interviews were carried out by six members of the IMPACT-ME research team, who were research assistants or PhD students, with specific training in in-depth interviewing. The majority of the parent interviews were carried out by a PhD student who was exploring the experiences of parenting an adolescent with depression. Data analysis for the present study was carried out after the report on adolescents’ experiences of taking part in the IMPACT trial had been published, and thus, the researchers did know the results of the earlier study when analysing and writing up the current study. The research team were aware of the potential bias this may have imposed on the data analysis, due to the potential risk of looking for ideas or themes that had been found in the earlier study. To counter this, we ensured that all interpretations were grounded in the data, by reaching consensus within the team on each of the themes in the analysis. Having conducted the analysis, we then explicitly went back to the adolescent paper and compared the findings, to see how their accounts were similar and different and report on these comparisons in the discussion of this paper (see the ‘Discussion’ section).

### Sample and data collection

The sample consisted of interviews with parents of 65 adolescents, who were distributed fairly evenly amongst the three treatment arms (BPI = 23, CBT = 20, STPP = 22); however, as the primary participants of the main trial were the adolescents, little demographic data was collected concerning this group of parents. When both parents were available to be interviewed, they were both invited to take part in the study and were interviewed together. Of the 65 families, the mother and father both took part in five cases, only the mother for 57 cases and only the father for three cases. The children of these parents had an average age of 14.75 years (range 11.38 to 17.84 years), and 65% were female.

Parents took part in semi-structured interviews using the *Experience of Therapy Interview* [15] at the end of therapy (36 weeks) and 1-year follow-up (86 weeks). The interview schedule (see Additional file 1) covered two key areas: the parents’ perspective on their child’s therapy and their perspective on taking part in the trial. The former has been reported elsewhere [18, 19], and the present study focuses on the analysis of data in relation to parents’ experiences of taking part in the research. The interview schedule provided open questions and a loose agenda for the key areas to be explored in the interviews, while also providing flexibility for the interview to focus on the area’s most pertinent to participants. Research assistants conducted audio-recorded semi-structured interviews, which had an average duration of 64 min (SD = 13.52). In order to gain as comprehensive a view as possible of their experiences of the trial, this study draws on data from both the 36-week and 86-week interviews, which were regarded as one account during analyses. The interviews were transcribed verbatim, anonymized and entered into qualitative analysis software, NVivo Version 12.0 [20].

### Data analysis

Data were analysed using framework analysis [21, 22]. Framework analysis is a data management and analysis approach which is integrated into the NVivo qualitative data analysis software and is suitable for studies with specific research questions, a pre-designed sample and a priori issues to investigate [21, 23]. The main a priori defined topics that we looked at were motivation to participate, acceptability of trial participation and understanding of different aspects of participation. Acceptability of trial participation was a focus of the interview schedule and as such was defined at an early stage in the research as a key area of interest for the research team. Other topics became of interest during the data collection phase of the research, where issues around motivation and variation in the way in which parents understood the research came up during the interviews—and as such, were defined prior to formal data analysis as key areas for further exploration.

In relation to these three a priori concerns, subcategories emerged from the content of the interviews. The second and third authors developed an initial coding framework in line with the aforementioned three main categories. One coder was a postgraduate researcher, and the second was a post-doctoral researcher; one of them was not clinically trained, and the other was a trained systemic therapist. To develop the framework, they coded the first 24 interviews together with the aim to develop a comprehensive, consistent and clear coding framework. They worked through each interview, line by line, assigning codes to each unit of meaning that told us something about one of the three a priori areas of interest for this study. Following this, they coded four interviews with this framework separately which were then compared to check reliability. To ensure that the coding framework was sufficiently comprehensive and reliable, the last author coded one interview using the framework and the feedback was used to further clarify the categories. The coding framework was finalized after coding these 29 interviews. The framework was then used to index and summarize the remaining interviews based on the coding framework.

Having coded all of the interviews into the NVivo software, the team then began the interpretation of the data coded to each of the three key issues: motivation, acceptability and understanding of the research—to make meaning from the range of experiences within each of these domains, seeking to identify similarities and differences between participants’ experiences. In addition, each case was coded on a global level in terms of the main motivation for participation, general acceptability of participation and the overall understanding/lack of understanding of participating in the RCT.

### Ethical considerations

The IMPACT and IMPACT-ME study protocols were approved by the Cambridgeshire 2 Research Ethics Committee, Addenbrooke’s Hospital Cambridge, UK (REC Ref: 09/H0308/137). Fully informed written consent was sought from both adolescents and their parents. Participants were identified having sought help at CAMHS and depression being considered as the main presenting problem for the adolescent. The assessing clinician would briefly describe the study to them and ask if they were happy to be contacted by a researcher from the trial team. If they gave verbal consent to be contacted by the trial team, the researcher would phone the parent and/or adolescent and explain the study in detail to them and post out the participant information sheet. There were separate, age-appropriate information sheets for younger (11–15) and older (16–17) adolescents, as well as parents. Following this, if the young person and (for those under 16) the parent both verbally agreed to take part, a screening assessment to assess eligibility for the trial would be scheduled, and at this point, fully informed written consent was sought. The researcher taking consent would go through the full details of the trial, including explaining the three treatment arms, using a pre-agreed description of each treatment, the randomization process and what involvement in the research would involve. Consent was re-affirmed verbally with participants at each research interview.

## Results

Findings are presented in three sections, following the three research aims of the study. In order to protect the confidentiality, identifiable details are excluded or disguised and participants have been assigned pseudonyms.

For seven of the 65 parents, the interview focused entirely on the parents’ views of their child’s therapy, and the interviews were ended before the interviewer could ask about the parents’ experiences of taking part in the research itself. For the remaining 58 parents, not all spoke about all aspects of experience covered in the framework, and so the number of parents reported in each section of the results varies.

Although the parents in this study were in one of three treatment arms, the focus of this study is on their experience of the RCT rather than of treatment itself. During the analysis, we were conscious to look for potential treatment arm differences in participants’ experiences, but the experience of parents did not appear to differ by treatment arm, so in what follows, results are reported without reference to specific treatment arms.

### Motivation for taking part in the trial

#### Conditional altruism

The most common motivation for participating in the study was to help others, with 17 of the 58 parents (29%) reporting this as their motivation. They reported wanting to help other people, contribute to the study and potentially help with the development of services and provision for young people with depression:

If any good can come out of this and if some more support can be put through, if the government get involved with this, so they can have more support within schools to try and lift the spirits of some of these kids then that’s what I’m happy with. (Ms Austin, mother of 16-year-old girl)

Six parents spoke about a feeling that participation in the study was something that ‘needs to be done’. This reflected their understanding of the need for research to help identify the most effective treatments for adolescent depression, and thus were accepting of being involved. Alongside this, parents described the motivation to help others, while also hoping for benefits for their own child.

#### Potential benefits to trial participation

Another common motivation for participating in the trial was to get help for their child and themselves. Fourteen parents spoke about their participation in the study in relation to getting treatment. The predominant theme seemed to be a relief at the trial offering some form of support:

I was kind of sort of at the end of my tether and I was just so pleased to be plugged into something, that’s kind of it. I keep, you know, I was very happy she got the CBT but if she got something else it wouldn’t have bothered me too much because I was just so pleased to get some kind of help. (Ms Toomer, mother of 16-year-old girl)

Ten parents reported their motivation was to get faster treatment than they would otherwise have been offered, while one parent reported that they felt they would be offered better treatment though the trial because ‘they’re [the therapists] gonna have to do everything by the books’ (Ms Heron, mother of a 16-year-old girl).

Parents also spoke about what they thought their child’s motivation for taking part in the trial was. Nineteen parents thought that money was the main motivation for their child’s participation (as they received a ‘thank you’ payment for participating in the research meetings). Seven parents reflected that being part of the study gave their child a sense of not being alone, and made the young people feel special:

For her she’s excited ‘cause she knows that she’s going to be part of history. (Ms Featherstone, mother of 16-year-old girl)

Thus, there was a range of reported motivations for participation including altruism as well as potential perceived benefits for the parent and/or child.

### Understanding of the trial

#### General understanding of the trial

Of the parents who spoke about their understanding of the trial, there was a similar proportion of parents who seemed to have a reasonable overall understanding of the study (17/36 parents; 47%) to those who seemed to have a general lack of understanding of the study (19/36 parents; 53%).

While eight parents demonstrated some understanding of the difference between research and therapy, seven parents seemed to be confused about the difference and more specifically the roles of researchers and therapist. For instance, one parent asked the research assistant ‘are you a doctor… maybe you are a psychiatrist or come from mental health?’ (Ms Sauter, mother of 14-year-old girl).

Of the 11 parents who referred to the objectives of the study, eight (73%) had a good understanding of the aims and objectives of the study, with all referring to the idea that the study was aiming to help professionals learn more about which treatments are helpful for depression:

So, my understanding is that the IMPACT study, you’ve basically, you’ve kind of followed people, or young people who have been in some kind of talking therapy, over the course of however long. So that is just gonna help understanding of which therapies work best for which types of individuals, right? (Ms James, mother of 17-year-old boy)

On the other hand, three parents seemed to be unable to remember or were confused about the objective of the study.

I think for me it was a research on how the session works for adults or… am I right? (Ms Bland, mother of 15-year-old boy)

With regard to consent, three parents gave the impression that they understood the process of informed consent, stating that they felt they had the right to stop participating or withdraw from the study:

You know, they said, ‘would you think of being involved in this?’ And I was kind of in two minds. I was thinking, if throughout, you know, as part of the randomization process he got the psychotherapy, I did kind of think that I might consider pulling out if I didn’t feel that was the best therapy necessarily for [my son] at the time. (Ms James, mother of 17-year-old boy)

#### Understanding of the treatment allocation process

Of the 24 parents who spoke about treatment allocation, 14 (58%) parents seemed to demonstrate a lack of understanding of the randomization process. This varied from stating that they did not know about randomization or could not remember it to feeling that it would have been better to have a choice between treatment arms, to those who stated that they believed the professional would choose the ‘right’ treatment for their child.

I thought I’ll leave it to the team, you know, well, I’ll leave it to when they’ve done the assessment. I trusted the assessment. I trusted it, that it would be the right one for her, you know, that it would be the right team for her. (Ms Monte, mother of 14-year-old girl)

In comparison, ten parents (42%) seemed to have a good understanding of randomization. These parents understood the necessity for randomization of treatment as part of a research study, the fact that there were three different treatments that their children could be randomized to and that all treatment options were potentially helpful.

I suppose again it’s the research, isn’t it? So, you know it’s kind of like we are guinea pigs in some ways and so it’s kind of like to me…you know you have to do it in a certain way you know and so there’s maybe not scope for those kind of more one-to-one things you know, more individual tension, erm really. I just think it’s the research that needs to follow certain ways of doing things and making it kind of a fair system. (Ms Heron, mother of 16-year-old girl)

Of the 17 parents that spoke about the treatment options, nine (53%) showed an understanding of the different treatment options. They spoke about the different treatments, in some cases remembering the names of them, but giving the impression that they had a sense of the differences between them.

I was actually quite pleased when I heard that he got the CBT... just because again I’d done some quick research into and because I think obviously we... were at a really desperate stage. It seemed to me that CBT – from what I’d heard and from friends that have done CBT - I had the impression that that was the one that got quite quick results if not necessarily long-lasting results. (Ms James, mother of 17-year-old boy)

In contrast, eight parents did not seem to have such a clear understanding of the differences between the treatment options, stating that they could not remember what they were and, in some cases, explicitly saying that they did not have a good understanding of the differences:

It was pot luck and since we didn’t really have a good understanding of the different kinds of therapy so we sort of thought, well... you know, I came back and spoke to [my daughter’s father] and we just sort of thought, well, because we don’t... she actually couldn’t care less at that point who she saw. (Ms Porter, mother of 17-year-old girl)

These findings indicate a substantial variation between parents with respect to how well (or not) they understood the research study.

### Acceptability

Of the 57 parents who referred to the acceptability of participating in the trial, 50 of them (88%) found participation to be generally acceptable, with seven (13%) finding participation to be generally unacceptable. Parents discussed the acceptability of the trial across a number of domains, including the process of randomization to a treatment arm, the research meetings and some specific issues around confidentiality, feeling pressured into taking part and financial incentives for participating. Each of these will be discussed in turn.

#### Randomization

Of the 23 parents who referred to the randomization process, 13 (57%) gave the impression of finding it to be acceptable. They reported that they did not mind that treatment was randomized as they were relieved to be getting help. Other parents stated that they trusted the professionals so felt able to leave treatment allocation in their hands, while one even found it preferable that the treatment allocation had been random:

I don’t think it made any difference to her at all, no, um I think that it’s such a new world for her, it didn’t make any difference. And even if someone had said to me which would you like, I wouldn’t have known what to choose. I really wouldn’t have known at all so in a way... probably the best thing. (Ms Adams, mother of 13-year-old girl)

However, eleven parents gave the impression of finding the randomization process unacceptable. Amongst the main reasons for this seemed to be an idea that a randomly selected therapy may not be what was best or most suited to their child, that the young people and parents should have the right to choose, and subsequent anxiety about whether they would be allocated to the right treatment or to a treatment that they deemed to be inadequate or unfavourable:

I would’ve liked it to be a little bit more tailored, like or at least be able to have the choice of the three. At the time, I remember thinking, I remember thinking, well, I’d prefer her to have, erm, cos I had the leaflet with the… I dunno where that is now! But there were three different choices, options and I don’t think she got the one that I thought sounded most helpful. (Ms Woods, mother of 13-year-old girl)

As demonstrated in the excerpt above, some parents were unhappy with the principle of randomization and specifically the lack of choice over the treatment their child received. Four parents spoke specifically about how they felt their child had been allocated to the wrong therapy and that the randomization process meant that the needs of their child would not be met appropriately.

#### Research assessments

Another key aspect described by parents was with respect to how acceptable (or not) the research assessments were. Twenty-three parents reported finding the opportunity for themselves and the young people to talk about their difficult experiences with a member of the research team a positive, helpful and therapeutic aspect of the study. For example, one parent described how completing the outcome measures for the trial had provided an opportunity for their child to express themselves and that this had helped them.

I do feel though that you know with doing the IMPACT it has helped him a lot. Erm, because I think like with all the questionnaires, that he’s done, it’s made him sort of understand a bit more about his own feelings. Because it is hard, you know, for children to express themselves. (Ms Baker, mother of 12-year-old boy)

Seven parents seemed to find the demands of participation manageable, or even enjoyable. The predominant feeling was that participating was not too demanding or ‘too onerous’ with meetings only ‘once every couple of months’.

It’s an hour out of my day and for you it’s the end of two years’ hard work… so for me, I just, yeah, didn’t think twice. I came because… you needed me to come. A lot of people won’t bother, and they won’t come and then you will have lost them and then, for me it’s an hour out of my morning so that’s fine, that’s cool. (Ms Thomas, mother of 12-year-old boy)

However, 21 parents reported finding the study burdensome. These participants spoke of feeling that participation in the study was time-consuming and intensive. The length of the meetings with the research assistants, particularly the initial, baseline assessment meeting (which typically lasted up to three hours), also seemed to be unacceptable to them, with phrases such as ‘too much all at once’ and ‘awful’ being used to describe the experience. Some parents spoke of the difficulty of going back over difficult experiences and the pain of talking about their child’s distress:

Yeah, and it is hard going back over it again, you know, it is, cause they were really upsetting times but I know that at the first interview, the first IMPACT interview I did, I was, you know, I did have a bit of a cry, you know, and I know and [my daughter] was like, had a box of tissues and so on. (Ms Creedon, mother of 17-year-old girl)

Parents also spoke of some specific criticisms of the study relating to the volume of questions that they had to answer, with some questionnaires seeming repetitive, irrelevant or tedious; limiting answers to time scales that felt too short or too broad; and the scoring scales seeming irrelevant, e.g. ‘0–10’:

It was kind of, you know, on a scale of (a) you know very poor going right through up to excellent or whatever erm... ‘how’s this been, how’s that been, how’s the other been?’ And yeah, that’s, that’s quite a difficult one to answer and also kind of complicated but sometimes you’ll read the situation and think I’m not sure this applies or does it apply, or I don’t know if it applies but I’m not sure how easy it is to get around that. (Ms James, mother of 17-year-old boy)

Other parents described finding the questionnaires interesting and some find that as the study progressed, the questionnaires helped with their understanding and awareness of their child’s depression:

Some of the questionnaires that I filled in made me understand a bit more about psychological issues. So, for example, people who suffer from depression, or any kind of low mood, I didn’t know that they always feel tired all the time, you know, and my daughter feels tired all the time. So, you know there are things like that, even though they didn’t say it in the questionnaire, I could read between the lines, where they were going with this. So, it gave me an insight to go and look up certain things, to work out why she was like she was… So, in some respects it’s helped me to tailor, the way that I look and treat her. (Mr Watterson, father of 14-year-old girl)

A positive aspect often spoken about by parents was with respect to the research assistants they had met with during their participation in the study. Thirty-one parents reported that they had had positive experiences with the research assistants, describing them as friendly, helpful, non-judgemental, understanding and professional. Research assistants gave the option for the research assessments to take place in the family home, which parents often spoke positively about:

I love the fact you come here. I have to say it sounds like a really small thing but actually particularly if you’re traipsing left right and centre to appointments with, with your daughter you know another appointment on top of that somewhere else would have just probably been the final straw so for it to at least be here it makes life an awful lot easier so that was a good thing. (Ms Adams, mother of 13-year-old girl)

While parents were often positive about their experiences with the research assistants, one issue that was raised was staff turnover, as the research assistants often changed over time. Eleven of the parents reflected that they did not mind changes in the research assistants, while 19 parents seemed to find changes in the research assistants less acceptable, stating that they liked having consistency and continuity in the research assistants that they met with. The advantage of having consistency with research assistants from the parent’s perspective was that it allowed for a relationship or rapport to be built up with them and with their child.

#### Issues with confidentiality

A small number of parents spoke about specific aspects of the study they did not find to be acceptable. The issue of confidentiality was discussed by four parents, who raised concerns about whether the research data would be confidential. Understanding that their data would be confidential was described as making participation acceptable for one parent. One parent expressed significant dissatisfaction with the information their child shared with the researcher not being shared with them as parents:

I want to know how my son is doing... because I think he’s doing ok and... but maybe just maybe he’s hiding it better from me... and then, if something happens to him, if he does kill himself, where do I go? I’ll come to you and I will blame you for it... because when I ask how he’s doing... you can’t give me the answer. (Ms Kowalski, mother of 16-year-old boy)

This reflects concerns around the research procedures, which would protect the child’s confidentiality, unless there were specific safety concerns.

#### Feeling pressured to take part

A specific issue with the trial raised by one parent was that they felt pressured into taking part in the trial:

It didn’t feel that we had much of a choice, to be honest. When we went along, initially I thought that you know but it was already like, well, this is the study, the IMPACT study and we were kind of like encouraged to do it. It was kind of like as if to say, well, you’re gonna get a lot more involvement in this and you know it’s gonna be…recorded and everything and all the plus sides of it which is good, but it just made you think, well, you didn’t have a choice! Well, we did have a choice but, you know, you’re kind of like pushed into it a little bit and that kind of thing. Erm…so you went along with it. (Ms Heron, mother of 16-year-old girl)

The perceived feeling of lack of choice over whether to participate in the study implied an issue of feeling pressurized to participate in order to access treatment for their child, due to lack of alternatives for seeking timely treatment. For another parent, an aspect of the study that they described as less acceptable was the financial incentives given to the young people for participating in the study:

My only thing about that is my one little criticism I have and I can understand is how they get paid...and I know I think we might and she got £40... and she didn’t even expect it, but she was like, she went out and bought a load of make-up... but obviously perhaps research has shown that is a motivating factor with young people, but I find that a little bit hard to take. (Ms Thomson, mother of 15-year-old girl)

This raises a specific question about the acceptability of ‘thank you’ payments for participants in research studies, which may be an important motivating factor for trial participation.

## Discussion

This study aimed to investigate parents’ experiences of participating in a clinical trial, in the context of a psychological treatment RCT for adolescent depression: the IMPACT trial [9, 10]. The study explored parents’ motivations for participating in the trial, their understanding of the trial and their perspectives on how acceptable they had found participating in the trial.

With respect to parents’ motivations for taking part in the trial, a wish to help others who might be facing similar situations was most commonly cited. Conditional altruism has been referred to as a primary motivating factor for trial participation in previous studies—that is, taking part in the hope it will help others, while also hoping they will benefit from it themselves [24]. These motivations are in line with the findings from a meta-synthesis of factors affecting recruitment of adults into depression trials [25]. These also overlap with the motivations reported by adolescents themselves for taking part in the same study [12], suggesting a shared motivation to participate in clinical trials between young people and their parents. Some parents’ motivation for participating in the trial was due to perceiving they would get faster or superior help than would have been on offer otherwise. Although all those who chose not to participate would still have been offered help at CAMHS, the concern about potentially long waiting lists may have impacted on the perceived choices that parents and young people had. This is an important reflection of overstretched CAMHS in the UK public healthcare system—meaning that for some of these parents, they felt they had little choice as they felt that the research would provide the quickest route to help for their child. Some parents also reported that for their child, their main motivation had been the ‘thank you’ payment for participating in the research assessments—an issue that was also mentioned by the young people who participated in the IMPACT study [12]. The use of payments for research participants has been controversial, with some authors arguing that such payments can be coercive [26]. Motivations for taking part in the study thus encompassed both an altruistic component, alongside perceived benefits for them or their child.

A basic ethical requirement for modern-day research is that participants should be fully informed as to what study participation will involve [27]. The present study raises some concerns about the extent to which participation of parents was truly informed, given that a substantial minority of the participants in this study did not appear to fully understand the study. While this may be a case of parents not remembering the details of the trial, consent was re-affirmed at each research meeting, suggesting that for some parents, they had not fully understood the trial information. Some parents appeared unsure of the distinction between the research and therapy and were unclear about the different interventions on offer through the study and the treatment allocation process. Several parents did not appear to understand randomization, an aspect of trial participation that other authors have identified as being difficult for participants to understand [28, 29]. The distinction between research and clinical care was not always clear to these parents, linking with the literature on ‘therapeutic misconception’, where participants believe that every aspect of the research is intended to be for their benefit [30]. Some parents in this study reported believing their child would be allocated to the right treatment for them, indicating an expectation for individualized care for their child. The issue of therapeutic misconception is cited as being more exaggerated in psychological trials compared with pharmacological trials, as such trials frequently involve testing interventions that have clinical equipoise and parents will often have expectations that the professionals conducting the trial are functioning with the child’s needs as their top priority [31]. In addition, in psychological trials, the distinction between the intervention (meeting a professional and talking together about your psychological well-being) and a research interview (meeting a researcher and talking together about your psychological well-being) may not be as easy to establish as it is in trials involving medication.

Despite some issues in parents’ understanding of the study, the majority found participation in the trial to be acceptable. Previous studies reported that participants frequently describe disappointment when allocated to the control group [32, 33], especially when parents have a preference towards one of the treatment arms [32]. In this study, parents rarely expressed a preference for one specific type of treatment, potentially due to the IMPACT trial involving three active treatment arms, which may have made the randomization process more palatable than in studies where participants may be allocated to a control or treatment as usual group. This was prominent in several of the parents’ accounts, who spoke about the relief of getting help, regardless of which of the treatments their child received. This may reflect parents having little preference amongst different types of talking therapies, as it may be difficult to understand the differences between them, especially for those without previous experiences of psychological treatment. Nevertheless, in this study, some parents did find the randomization process to be unacceptable, due to fears about their child receiving a treatment that may not have been right or best suited to their child.

The research assessments were also frequently referred to, and parents again varied in how acceptable they found them. While some reported that they were not demanding and some even found them helpful or therapeutic, others were more concerned about the burden the assessments placed on them and/or their child. The number of questionnaires and length of the assessments were referred to, with the baseline research assessment seeming to be the least acceptable. These assessments were typically the longest, and there was a sense of it being too much to deal with at a very difficult time in their lives. In the IMPACT study, a lengthy battery of questionnaires and interviews had been included in this baseline assessment. However, after the study began, this feedback from families led the trial team to reduce the participant burden. Parents often spoke positively about the research assistants who they had met with throughout the study. The sense of having friendly and non-judgemental research staff, who were flexible to make the appointments convenient for the family, was striking—and were potentially important in making the study generally acceptable to parents. This is an issue which has not been widely commented on in the literature to date, and may be worth considering, especially as trials are increasingly moving to remote or online data collection where face-to-face contact with the research team may be reduced [34].

There were also specific concerns about the study raised by a minority of parents. The issue of confidentiality was raised, with one participant expressing concerns about not being kept informed about their child’s progress from the research assessments. We are unaware of previous studies that have cited this finding, but is an important consideration when working with parents of children where there are specific concerns around risk. This raises an issue of working with adolescents, in striking the balance between the young person’s right to confidentiality and the concerns of the parent about their child’s safety. However, this is likely to be an extension of what may occur in treatment with young people outside of a research study, where parents feel they want to know what is happening in their child’s therapy.

### Recommendations for trial design

This study suggests that most parents identify clear motivations to take part in clinical trials in the field of child and adolescent mental health and overall found the research process acceptable. This study shows us that trial participation can be a positive experience for parents. Positive interactions with research staff and flexibility of researchers are important considerations from the perspective of parents participating in clinical trials. Including young people and parents in the recruitment of researchers is one way that their views can be valuable, to ensure that trial staff have the qualities deemed important from the perspectives of parents, as this was a crucial aspect of acceptability in this study. Indeed, we included parents and young people on interview panels in the recruitment of staff for the IMPACT-ME study, and this was hugely important to ensure that the team were people who participants would feel comfortable with during interviews, given the sensitive nature of the project the team were responsible for.

There are however important considerations for the design of future trials. First is the issue of how to ensure that consent is fully informed. In this study, parents did not always have a clear understanding of the purpose of the clinical trial, the rationale for randomization or what participating would involve. Parents were making a decision about whether to participate in the trial at a very stressful point in their lives, where their primary concern was to access help for their child’s mental health problems. One reason for taking part was to get quicker access to help for their child. However, even when clinical resources may be scarce, and there is the possibility of long waiting lists for treatment, participants should not feel pressured into taking part in studies and they should feel it is a choice as to whether to take part.

Future studies should seek to find better ways of communicating with potential participants the aims, objectives and research process—as existing information sheet design appears insufficient for communicating such complex information. A possibility might be, for example, to arrange a meeting with each participant young person and parent, to talk through the information sheet, to ensure that the issues have been understood and to discuss pros and cons, rather than just to give the sheet to the participant and give them a chance to raise questions. Potential participants may not know that they have not understood the study design, or how to frame a question or concern, without help from someone more familiar with the research and treatment conditions. Alternatively, researchers could seek to establish more creative ways of communicating study information to participants. For instance, short films or animations may be a more engaging way to communicate key information about the study to participants [35], compared with the existing information sheets, which can be dense with information and may overwhelm some participants. Patient and public involvement in developing such resources are crucial to ensure that study materials are accessible for people with varying degrees of literacy and from different cultures. Indeed, this approach has been found to ensure that research information and tools are user-friendly and accessible to participants [36]. However, issues remain about the lack of systematic reporting about patient and public involvement in research [37]—which limits the way in which researchers can build cumulative knowledge about the most effective ways of involving stakeholders in research.

In the 10 years since the IMPACT trial was designed, there has been an increased emphasis on public and patient involvement (PPI) in research. This study reflects the valuable insight that parents can provide in the design and delivery of research. Patient and public involvement should be included at all stages of the project, such as reviewing trial materials as part of developing research protocols and seeking ethical approval [38] and extend into the training of researchers. For instance, parents could be involved in training research staff to ensure they communicate effectively with participants and understand the priorities and needs of potential research participants.

### Strengths and limitations of this study

A strength of this study is its relatively large sample size, based on in-depth interviews with parents about their experiences of participating in a clinical trial. Although qualitative studies often benefit from having smaller sample sizes, to allow detailed analysis of individual experiences, having a larger sample size makes it possible to include a broader range of experiences and is recommended in framework analysis [21]. The study used semi-structured interviews with parents, allowing in-depth exploration of the topics most pertinent to participants and openness to the unexpected. While this allows the interviews to be participant-led, a limitation of this approach is that participants were not systematically asked about all elements of the study. It is therefore possible that some important opinions on specific aspects of the study may not have been expressed. The sample comprised only those who had already agreed to participate in the trial. It is unknown how representative these findings are of those parents who declined to participate in the trial or those who withdrew from the study—who may have quite different perspectives from those represented in the present study. A further limitation of the study is that limited demographic information was obtained from parents, and further, data on whether the parents had previously taken part in clinical trials, which would influence their understanding and perspectives of trial participation.

Finally, this study emphasizes the importance of PPI in all aspects of trial design and delivery. We note that the IMPACT trial was designed over a decade ago, when there was less emphasis on the inclusion of PPI, and funding bodies did not routinely require applicants to incorporate PPI work into their proposals. Since this time, there has been an important shift, and we hope that learning from this study can help others to consider the range of ways in which PPI can benefit research design and conduct.

## Conclusion

This study has helped to explicate parents’ motivations, benefits and burdens of participating in a trial. This study shows that the IMPACT trial was generally acceptable to parents, and the aspects of the study that parents had an issue with largely overlapped with those of the adolescents reported in a previous study [12]. Nevertheless, there were indications that not all parents gave fully informed consent to take part in the study, and there were a number of elements of the research design which some parents were not happy with. Together with the earlier study looking at the experience of young people taking part in the same clinical trial [12], these studies provide a wealth of information that can help to inform future trial design, to ensure that trials for young people in the field of child and adolescent mental health are acceptable, ethical and minimize the burden to both young people and their parents.

## Supplementary Information

**Additional file 1.** : Parent interview schedule.

## Data Availability

The datasets generated and/or analysed during the current study are not publicly available as consent was not sought from participants for data sharing.

## Abbreviations

BPI: Brief Psychosocial Intervention
CAMHS: Child and Adolescent Mental Health Services
CBT: Cognitive behavioural therapy
IMPACT: Improving Mood through Psychoanalytic and Cognitive-Behavioural Therapy
IMPACT-ME: IMPACT-My Experience
RCT: Randomized controlled trial
STPP: Short-term psychodynamic psychotherapy
