# Supplementary material for: Parents’ understanding and motivation to take part in a randomized controlled trial in the field of adolescent mental health: a qualitative study
Source: Trials. 2020 Nov 23;21:952. doi: 10.1186/s13063-020-04857-3 (PMC7684724; doi:10.1186/s13063-020-04857-3)
Supplement: Supplementary file 1 — Additional file 1. : Parent interview schedule. [file 13063_2020_4857_MOESM1_ESM.docx]

**Additional file 1. Parent interview schedule**

| **Experience of Therapy Interview Schedule - Parent/Carer** |
| --- |
| 1. **The difficulties that have brought the young person into contact with Child and Adolescent Mental Health Services (CAMHS)**  - Can you tell me how your son/daughter came to be referred to the CAMHS service? - What was going on for him/her at the time? - In what ways did these things affect your family's life at the time? |
| 1. **The parent’s understanding of those difficulties**  - How do you make sense of what was going on for your son/daughter at the time? - Can you tell me the story of how things came to be the way you described? |
| 1. **Change**  - Compared to about a year ago, how has your son/daughter been feeling/experiencing things? - Compared to then, what is similar or different for you as a parent? - In thinking about these changes you have just mentioned, what are the things that contributed to these changes? What has been helpful/unhelpful? - In relation to how things began/what was going on for your son/daughter at the time, do you see things differently now to how they seemed at the time? (How/why?) - Do you think your son/daughter sees things differently now? (How/why?) |
| 1. **The story of therapy**  - What was your involvement with [name of clinic]? - What ideas did you have about therapy before your son/daughter’s therapy began? - Can you tell me the ‘story’ of your child’s therapy as you see it? - Are there any specific moments or events that you remember about your son’s/daughter’s therapy? - Can you tell me about the ending of your child’s therapy? - What was it like for you knowing that your child’s therapy was a time-limited intervention? - Overall, how did it feel to have your son/daughter in therapy? What do you think it has been like for him/her overall? |
| 1. **Evaluating therapy**  - What were the most helpful things about the therapy for your child? - What kinds of things about your child’s therapy were unhelpful, negative or disappointing? - Was medication for your child ever discussed? If your son/daughter was starting therapy again, what would you like to be different? - What do you think were the most helpful things about your own involvement with the therapy? - What kinds of things about your involvement were unhelpful, negative or disappointing? - Do you feel that your experiences of your son’s/daughter’s therapy/your own involvement with [name of clinic] have affected your views now about how things began/what was going on at the time when your son/daughter was first referred to the [name of clinic]? (How/why?) - Do you think that your son/daughter would see it the same way? (How/why?) |
| 1. **Involvement in research**  - I'd like to ask you a few questions about what it has been like being involved in the research side of the IMPACT study. Can you tell me about your experience of being involved in the research side of things? - When you and your son/daughter initially joined the IMPACT study, your son/daughter was allocated to one of three treatments on a random basis. Looking back, how do you feel about that process? Did you have a view on which of the three you hoped to get/not get? How do you think your son/daughter felt about it? Do you think it had any effect on your son’s/daughter’s therapy? - Can you tell me a bit about the regular meetings with the research assistants?   *(Possible prompts: What has it been like having those meetings? Have you met different research assistants? How did that feel like for you? And for your son/daughter? Did you ever talk about those meetings in your therapy? What was it like to attend research meetings at different points in time while your son/daughter was still receiving therapy? And how do you feel now about attending research meetings after the therapy has ended?)*   - Overall, what’s it been like for you/your son/daughter to have had therapy as part of a research study? - Do you have any suggestions for us regarding the research? |

*Note: This study used data from section 6 of the topic guide only*
